# Laser Mechanized Physicochemical Characterizations on the Surface of Implants and Dental Abutments

**DOI:** 10.7759/cureus.84150

**Published:** 2025-05-15

**Authors:** Claudia Penélope Mora López, Hector Tellez Jimenez, Jorge Humberto Luna Domínguez, Sergio Eduardo Chávez García

**Affiliations:** 1 Faculty of Dentistry, Autonomous University of Tamaulipas, Tampico, MEX

**Keywords:** dental implants, grade v titanium, laser-lok, macrostructure, microstructure

## Abstract

Introduction

Dental implants have revolutionized dentistry, offering effective solutions for the rehabilitation of masticatory function and dental esthetics. BioHorizons Laser-Lok dental implants are known for their innovative surface, which uses laser technology to create microstructures in titanium, improving interaction with bone and periodontal tissue.

Objective

To evaluate the microscopic physical characteristics and elemental composition of titanium implants grade V.

Material and method

This descriptive and observational study evaluated physicochemically grade 5 purity titanium implants: Tapered (BioHorizons) Bone level Laser-Lok resorbable blast textured (RBT) titanium implant by scanning electron microscopy and energy dispersive spectroscopy. The sample analyzed consisted of a single implant unit corresponding to the aforementioned model. The samples were subjected to microscopic analysis for topography. The microgrooves of the implant neck and the frontal view were analyzed for microphotography and proceeded to their physical description.

Results

Defects were observed in different areas of the tooth abutment surfaces. The minimum and maximum size of the microgaps ranged from 0.5 μm to 5.6 μm. In addition, defects were detected throughout the implant-abutment joint that may ultimately affect the size of the microgap after connection. Conclusion. Laser-Lok biohorizons implants present an ideal macrostructure and microstructure for the placement process and primary stability, favoring biomechanics. In addition, due to their specific organization and reduced distance between the grooves, they allow adequate cellular organization and differentiation.

## Introduction

Dental implants are medical devices used in dentistry for the replacement of teeth lost or extracted due to decay, fractures, and infections. These devices are designed to restore chewing function and improve the esthetics and speech of a person with missing dental organs. One of the main challenges to the long-term success of implants is peri-implant disease, such as peri-implantitis, which affects the health of the tissues surrounding the implant and can compromise its stability and functionality [1]. Osseointegration, the key process in which the implant fuses with the bone, is fundamental for the success of the treatment; however, factors such as the type of material, the design and the surface of the implant can considerably influence this process, due to the wear and release of metal ions that affect its connection. Some studies have shown variations in their topographic surfaces, titanium being the most used material in implants [2].

The use of titanium and titanium-based alloys has increased significantly in the science of dentistry due to the increasing use of osseointegrated implants. In addition, specialists in the field have been encouraged to use these metals because of their physicochemical properties: biocompatibility, low density, low thermal conductivity, resistance to withstand high static and cyclic stresses of the masticatory system, low weight, low cost, and good corrosion resistance [3,4]. The main alloy used is commercially pure titanium, which is available in four grades numbered 1 to 4, according to purity and processing oxygen content [5,6]. These grades differ in corrosion resistance and ductility, with grade 4 being the most commonly used for dental implants [6,7]. On the other hand, there is the Ti-6Al-4V alloy, called titanium grade 5, which is widely used in orthopedics, however, it can also be used in dentistry because it is biologically acceptable [8,9].

For the two main alloys used to manufacture implantable devices, namely commercially pure titanium, cpTi and Ti-6A1-4V, the surfaces are mainly composed of TiO2 oxide [10]. Corrosion resistance and biocompatibility are directly related to the passive oxide layer formed on the surface of titanium and its alloys [3,11]. This oxide layer is 4 to 6 nm thick and contains hydroxyl groups in addition to the oxide. Titanium implants usually have their surfaces modified after their initial fabrication to ensure that the oxidation is uniform and that any contamination is removed [12]. The resulting surfaces have enhanced biological characteristics and promote cell adhesion and proliferation processes, which contribute to bone union [13,14].

Biohorizons implants are made of grade 5 titanium and have surface treatments such as Laser-Lok and resorbable blast textured (RBT) to promote osseointegration. These implants have a bond with the peri-implant connective tissue because they act as an effective barrier against apical migration of the epithelial junction. They are characterized by the use of microchannels to create a physical-functional junction that allows bone stability and reduces crestal bone loss [15]. The objective of the present study was to evaluate the microscopic physical characteristics and elemental composition of grade 5 titanium implants.

## Materials and methods

This experimental, descriptive, and observational in vitro study evaluated physicochemically grade 5 purity titanium implants: Tapered (BioHorizons) Bone level Laser-Lok RBT titanium implant by scanning electron microscopy (SEM) and energy dispersive spectroscopy. The analyzed sample consisted of a single implant unit corresponding to the aforementioned model.

The selection of a single unit was due to the study’s objective of performing a specific characterization of the design and surface of this commercial implant, rather than a comparative or population-based statistical analysis. Since these devices are industrially manufactured under strict quality and uniformity standards, homogeneity between units from the same batch or commercial reference is assumed. Therefore, a representative sample of a single unit provides reliable and replicable information on the product's physicochemical properties. The samples were removed from their packaging using sterile metal tweezers and prepared with a carbon cover and placed on the electron microscope stage (JEOL JSM-6460LV), and were subjected to microscopic analysis for topography.

This study was reviewed and approved by the Research Ethics Committee. Since this was an in vitro analysis of commercial medical devices, with no human or animal subjects involved, the committee determined that informed consent was not required, in accordance with the ethical provisions established by the institution and international guidelines for biomedical research.

The microgrooves of the implant neck and the frontal view were analyzed for microphotography and proceeded to their physical description. Subsequently, the implants were subjected to energy dispersive spectroscopy (EED) for their chemical composition analysis by means of a random mapping in which the implant was divided into three zones: 1) Implant body, between crest and crest of the implant strings for further analysis of the structure and composition in different areas of the string surfaces, 2) neck, straight and with micro threads, and 3) apex, dome, without hole or grooves (Appendices: Figure 8).

For the selection of the dental implants included in this study, the following inclusion criteria were implemented: titanium implants with grade V alloy, implants with surface treatment by laser ablation (Biohorizons) bone level Laser-Lok/RBT and implants with standardized dimensions of 3.8 × 10, this to guarantee the uniformity of the samples in terms of geometry. In addition, the following exclusion criteria were established to ensure the homogeneity of the samples and to avoid possible biases in the results: pure titanium implants, implants with other surface treatments, and implants made of materials other than titanium. Finally, the exclusion criteria include implants that have been damaged during the microscopic analysis process or that have been damaged in energy dispersive spectroscopy.

Data analysis

The analysis of the data obtained was qualitative in nature and was based on the detailed observation of the images generated by SEM, complemented with the results of energy dispersive spectroscopy (EDS). A descriptive morphological characterization of the implants was performed, identifying structural aspects such as the general body shape. In addition, the compositional characterization obtained by EDS made it possible to identify the elements present on the surface, although elemental quantification was not performed, given the descriptive approach of the study.

## Results

On scanning microscopy of the Tapered Biohorizons Laser-Lok implant, a tapered cylindrical body was observed, with an internal hexagon connection, and its thread type was an inverted abort V-shape. The distance between threads ranged from 600-800 µm. In the microchannels of the implant neck, the distance was 16-18 µm. The implant collar was precision-designed and has been shown to stimulate oblique connective tissue attachment much like that of the natural tooth.

Topographic results

**Figure 1:**
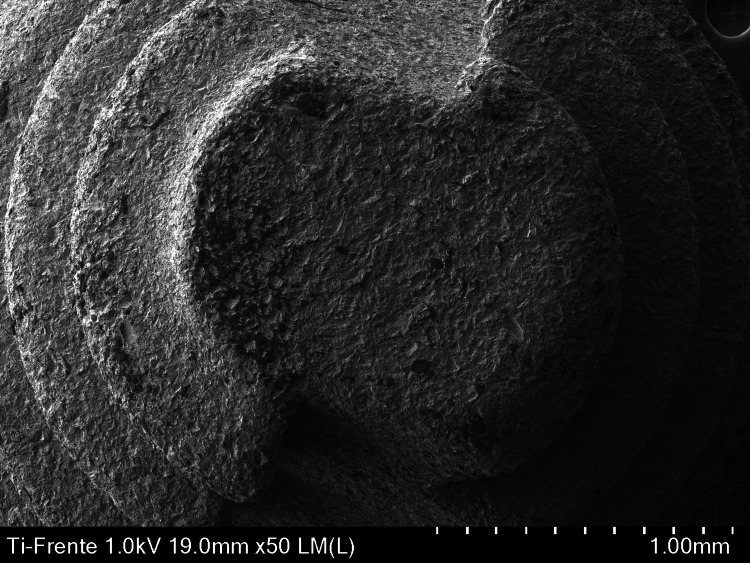
Anterior micrograph of the apical area of a Biohorizon Laser-Lok dental implant captured with a scanning electron microscope (SEM) at 50×.

Figure 1 shows a high resolution of 50×, in which a dome without grooves is visualized, allowing the texture and details of the implant surface to be observed. The microstructure is visible, and the surface shows variations in roughness, suggesting an optimization to favor osseointegration.

**Figure 2:**
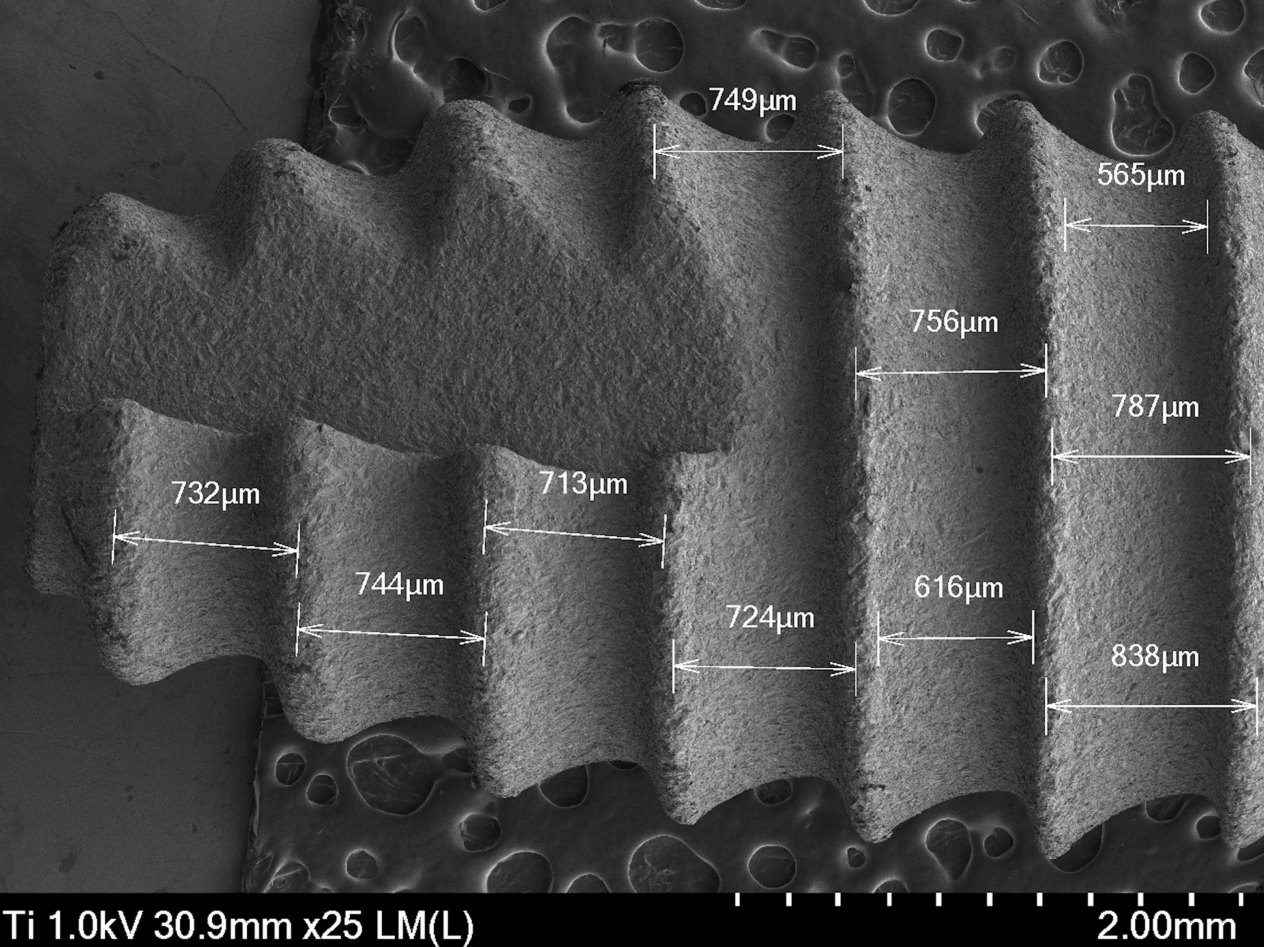
Micrograph of the apical lateral area of a Laser-Lok Biohorizons dental implant captured with a scanning electron microscope (SEM) at 25×.

At the apex, the implant surface presents a microstructured texture, with visible microchannels that facilitate interaction with the bone tissue (Figure 2). The roughness is visible, suggesting an optimized design to promote osseointegration. The distances between the strings or microstructures are crucial. The spacings between the microchannels or roughness peaks can be measured. These distances, which can vary, are fundamental to understanding the mechanics of bone integration, on average ranging from 732 to 838 µm.

**Figure 3:**
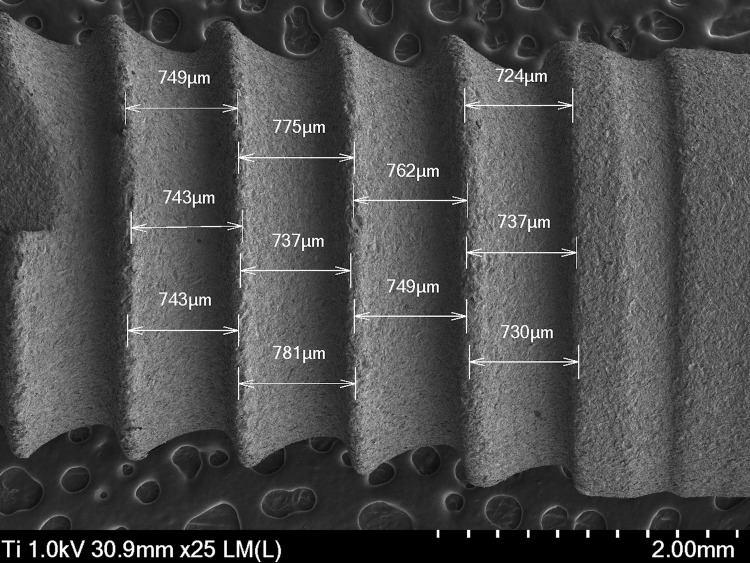
Micrograph of the body area of a Laser-Lok Biohorizons dental implant body captured with a scanning electron microscope (SEM) at 25×.

Figure 3 presents a wide view of the implant body, allowing one to observe its general design and surface characteristics in detail. At 25×, variations in roughness can be seen, which contribute to cell adhesion and bone growth. The texture can be uniform or present areas with different patterns that favor interaction with the surrounding tissue. Any irregularities on the surface, such as small protuberances or depressions, can be highlighted, since these characteristics can influence the behavior of the implant within the tissue. The distance between strings varies from 724 to 781 µm, with the distance in the middle part of the implant body being larger.

**Figure 4:**
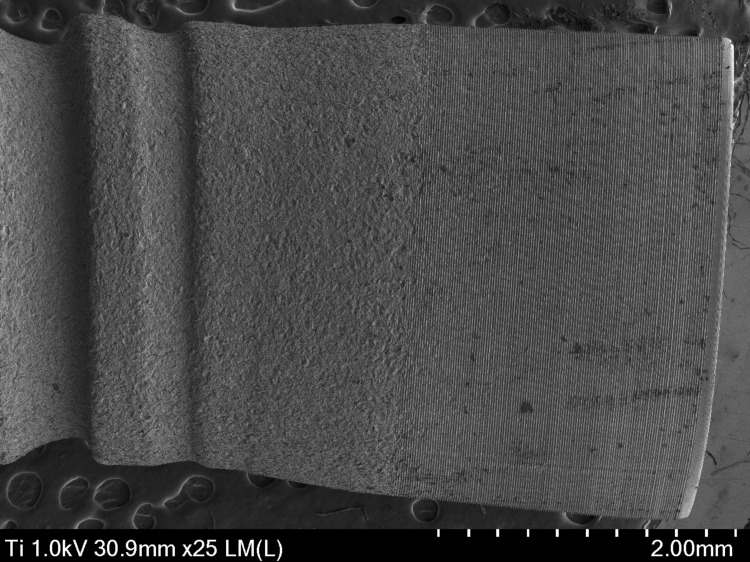
Micrograph of the neck area of a Laser-Lok Biohorizons dental implant captured with a scanning electron microscope (SEM) at 25×.

Figure 4 provides a detailed view of the implant neck, which is the critical region where the implant meets the soft tissue and bone. This area is crucial for its stability and integration. The surface of the collar has a texture that may be slightly different from that of the implant body. Often, a microtexture is observed that facilitates the adhesion of the surrounding soft tissues.

**Figure 5:**
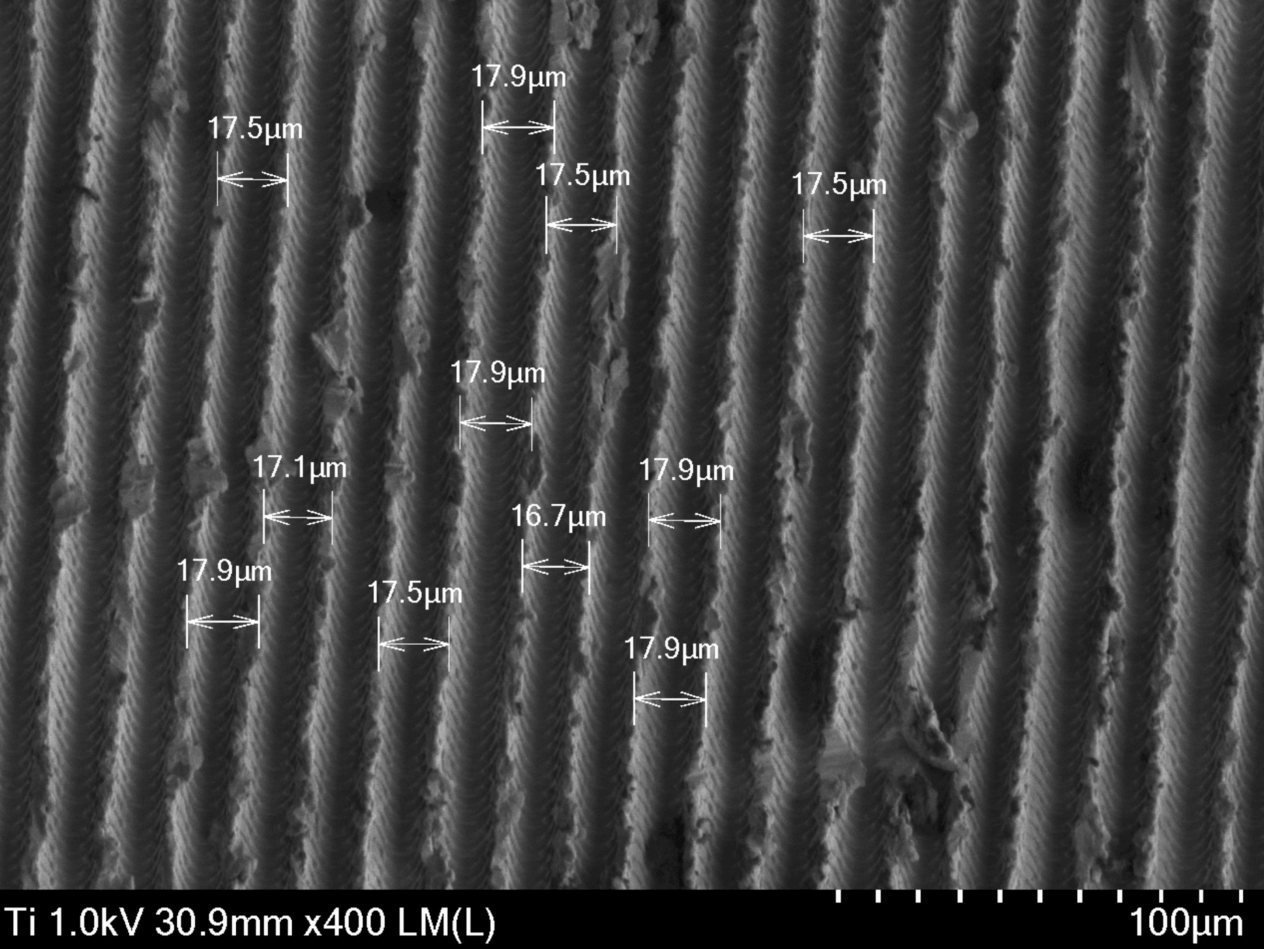
Micrograph of the neck area of a Laser-Lok Biohorizons dental implant captured with a scanning electron microscope (SEM) at 400×.

The collar may show a more subtle roughness pattern, designed to optimize the connection with the periodontal tissue. Microstructures, such as small indentations or canals, are visible and can influence biocompatibility, generally having a thinner design than the body of the implant, which can be seen in Figure 5. The shape and diameter of the collar ensure adequate insertion and stability, with microgrooves and a uniform thickness of 16.7 to 17.9 µm.

Energy dispersive X-ray (EDX) results

**Figure 6:**
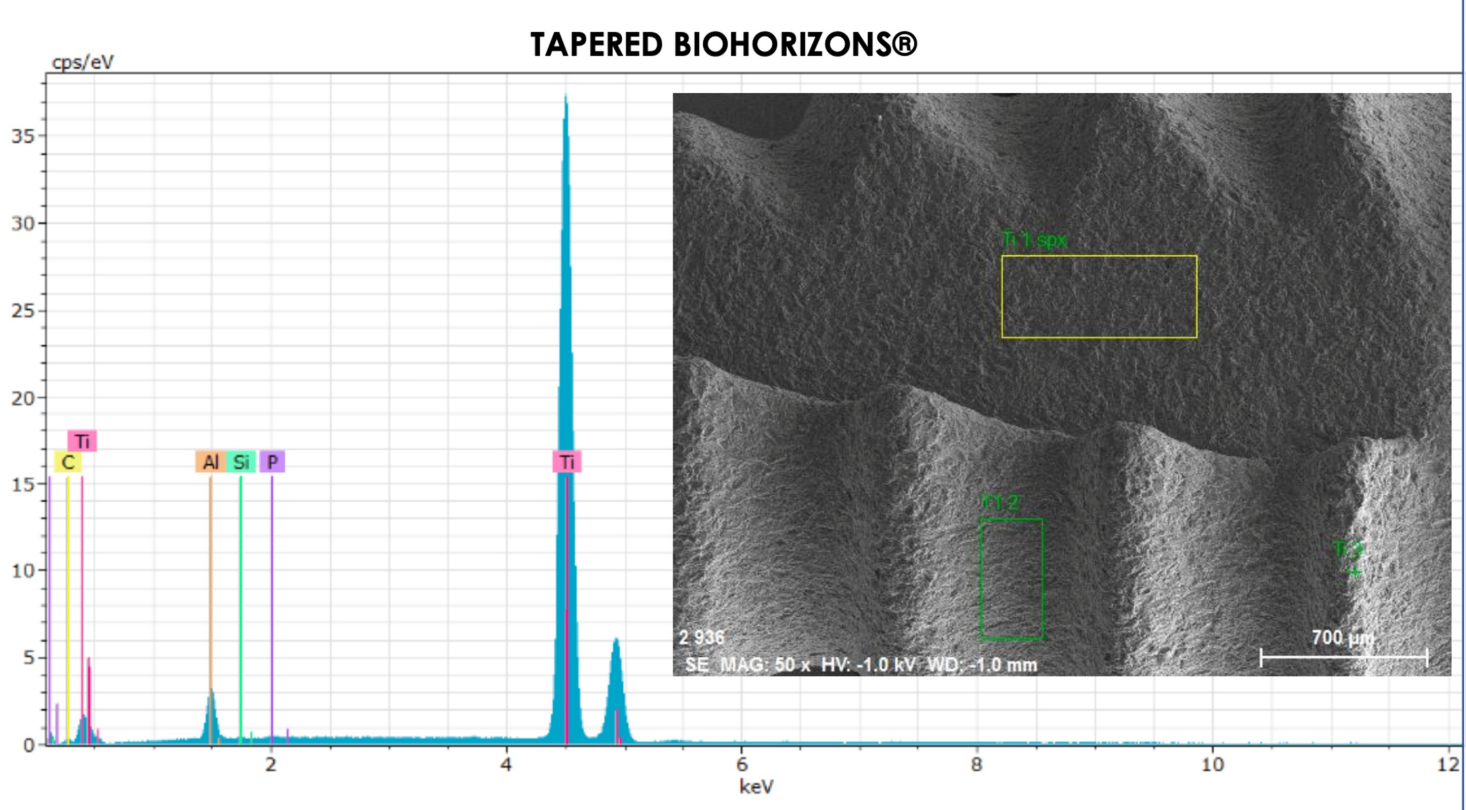
Energy dispersive spectroscopy of the titanium implant body. The observed elements are: titanium, phosphorus, aluminum, silicon, and carbon.

By means of EDS, the following elements of the periodic table (Table 1) were distinguished in the area of the implant body (Figure 6).

**Table 1:** Results of EDX analysis of the body area sample of a tapered Biohorizons implant. EDX: Energy Dispersive X-ray

Element	AN	Series	(wt.%)	orm. wt.%	orm. at.%	(1 sigma)
Carbon	6	K-series	1.219472	1.198222	4.428786	0.347801
Aluminum	13	K-series	5.46845	5.373158	8.840758	0.295053
Silicon	14	K-series	0.04856	0.047714	0.07542	0.029864
Phosphorus	15	K-series	0.145678	0.14314	0.20516	0.034759
Titanium	22	K-series	94.89133	93.23777	86.44988	2.651749
Total	-	-	101.7735	100	100	-

**Figure 7:**
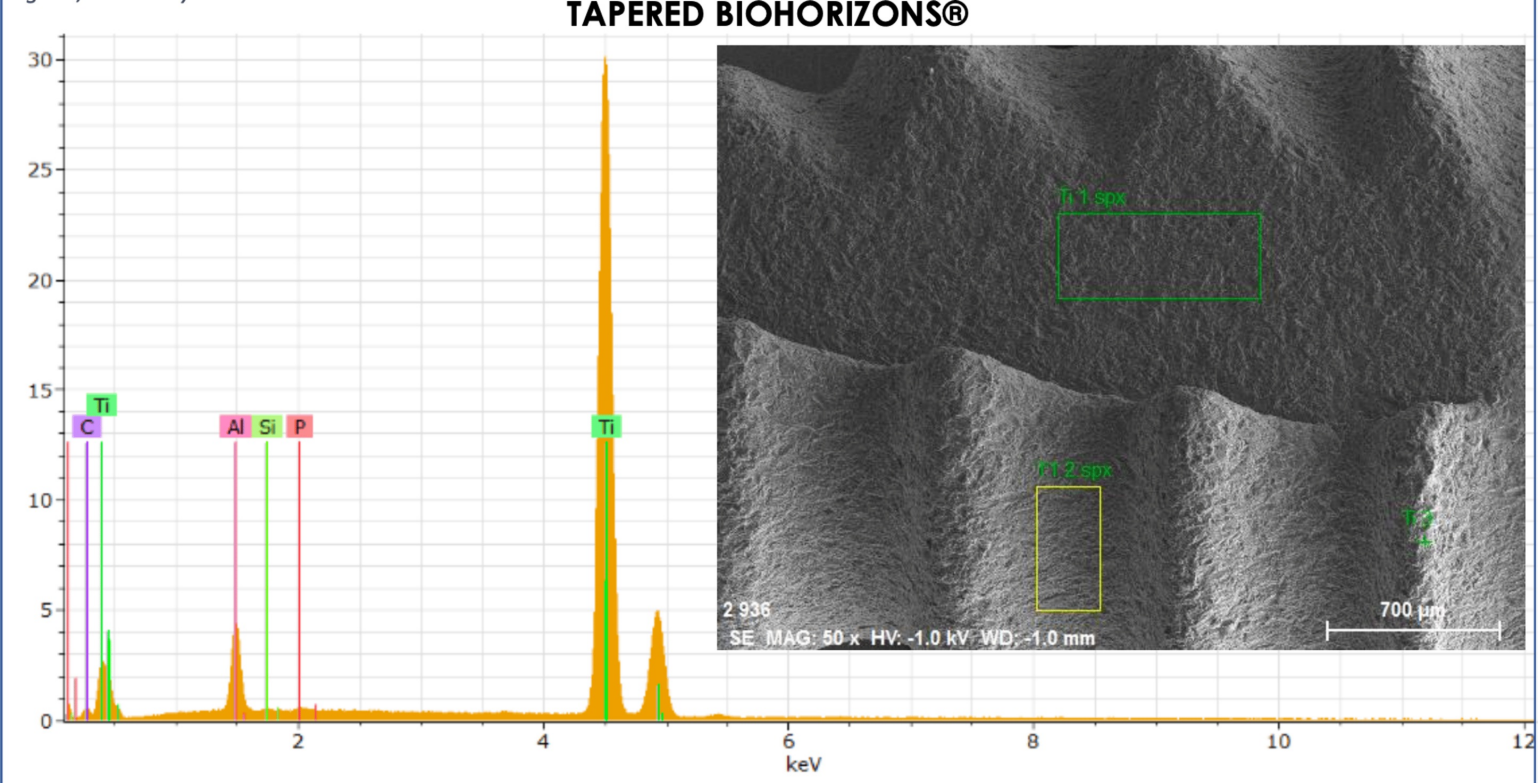
Energy dispersive spectroscopy of the inter-string valley of the titanium implant. The elements observed are: titanium, phosphorus, aluminum, silicon, and carbon.

**Figure 8:**
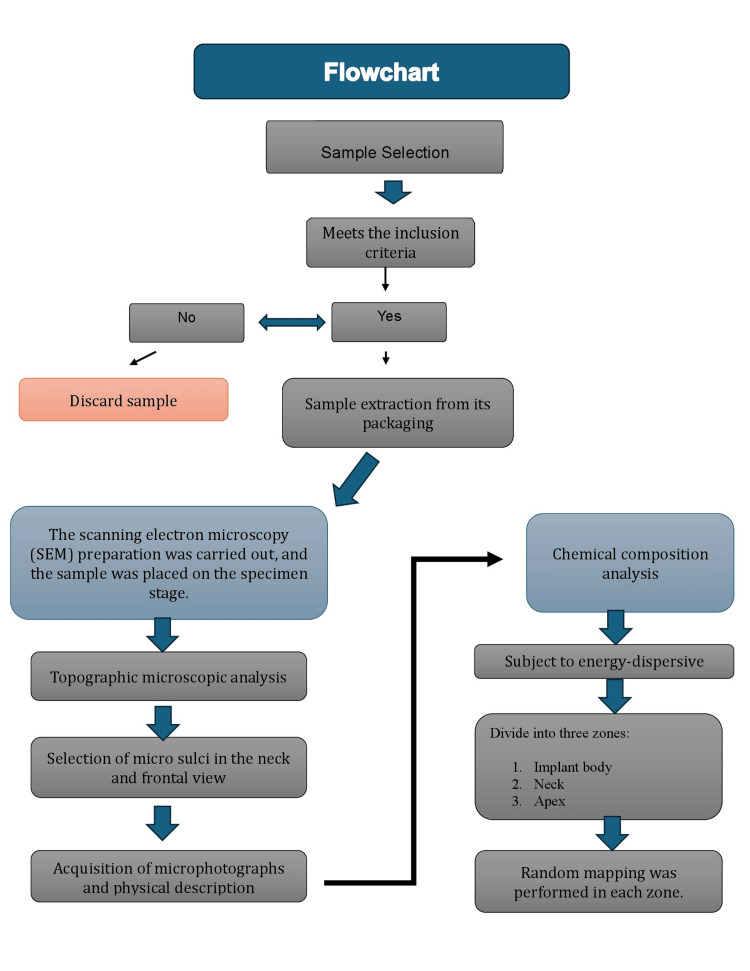
Flowchart The characterization of the implant is defined step by step.

In the spectrum analysis, the valley between the strings of the titanium implant was analyzed (Table 2), of where copper resulted from the analysis of the plate where the implant was placed (Figure 7).

**Table 2:** Energy dispersive X-ray (EDX) analysis results of the Biohorizons tapered implant valley sample.

Element	AN	Series	(wt.%)	orm. wt.%	orm. at.%	(1 sigma)
Carbon	6	K-series	35.56838	34.90859	4.428786	0.347801
Aluminum	13	K-series	23.98994	23.54492	8.840758	0.295053
Titanium	22	K-series	31.83317	31.24266	0.07542	0.029864
Copper	29	K-series	7.540186	-	0.20516	0.034759
Gold	79	K-series	2.958401	93.23777	86.44988	2.651749
Total	-	-	101.8901	100	100	-

## Discussion

Although dental implants have a long durability, they can suffer bone loss and affect their osseointegration, which can lead to implant failure. Therefore, one of the most important challenges in the field of oral implantology today is to prevent bone loss around osseointegrated and functional implants over the years. In order to try to correct this dilemma, different methods of implant surfaces have been designed to allow a correct peripheral sealing of the soft tissues in the cervical area of the implant [16].

Most dental implant surfaces that are available are sandblasted and/or acid-treated. These manufacturing methods create random surfaces that vary from point to point on the implant, in addition to generating an alteration in the cellular reaction that comes in contact with the surface. Although random surfaces have demonstrated greater osseointegration than machined surfaces, Laser-Lok surfaces have also been shown to be effective for soft tissue attachment by different microscopies.

Laser microtexturing is one of the surfaces that has been developed in recent years for use on implants and implant abutments. In a systematic review evaluating different types of implant surface treatments that have an impact on the biological seal between the gingiva and the abutment, it was found that surface treatment based on the creation of laser microgrooves can provide a benefit in the attachment of the connective tissue to the abutment. Weiner et al. studied for the first time in an animal model the effect of laser microtextured collars on bone, connective tissue, and surrounding epithelial cells, concluding that this treatment stimulated bone and soft tissue attachment, facilitating the development of a correct biological width [17].

On the other hand, Nevins et al. analyzed human histological samples demonstrating the efficacy of laser-created microgrooves in implant collars to achieve a direct connection of the connective tissue to the implant, thereby inhibiting apical migration of the junctional epithelium [18]. In addition, Pécora et al. observed radiographically a bone loss of 0.59 mm in laser-treated implants with the topography described at the level of the implant neck, in which there was a coincidence in our study due to the same characteristics observed in the SEM showing a more subtle roughness pattern, designed to optimize the connection with the periodontal tissue [19].

Microstructures, such as small indentations or channels, are visible and can influence biocompatibility. The shape and diameter of the neck are important to ensure proper insertion and stability. Chemically, the implant surface consists of a thin layer of titanium oxide, the cleanliness of which is considered a basic requirement for achieving osseointegration. The chemical composition of the layers includes the amounts of titanium (Ti), oxygen (O), silicon (Si), and carbon (C) in all samples. In a chemical analysis by Muñante-Cardenas, the presence of these elements was demonstrated; on our part, the same was obtained with the consideration of elements such as phosphorus (P) and gold (Au) [20].

The current study has certain limitations that should be taken into account when interpreting the results. Since this is a descriptive analysis focused on the topographical and compositional characterization of BioHorizons Laser-Lok implant surfaces, the results are not applicable to other implant systems with different surface treatments. In addition, the present study was performed only on implants from a single producer, which limits the ability to make comparisons between different brands that could broaden the understanding of technological variations. Although the methodologies used, such as SEM and EDS, provide detailed information on the microstructure and chemical composition, they do not allow assessment of the biological response and in vivo clinical behavior of the surfaces studied. Therefore, further investigations, including biological and clinical analyses, are recommended for a more complete evaluation of the performance of this type of implant.

## Conclusions

The Laser-Lok Biohorizons implants presented an ideal macrostructure and microstructure for the placement process and primary stability, a key factor for the success of the implant in the clinical environment, giving it greater durability. On the other hand, its chemical composition presented a homogeneous composition in its structure and a topography that made cell colonization feasible, which are indicators that the implant is biocompatible with the human organism. However, although the in vitro results are promising, further in vivo studies are recommended to evaluate its impact in clinical practice in a safe manner.
